# A Good Wife

**Published:** 1878-04

**Authors:** 


### A GOOD WIFE.

In the eighty-fourth year of his age, Dr. Calvin Chapin wrote of his wife: "My domestic enjoyments have been, perhaps, as near perfection as the human condition permits. *She made my home the pleasantest spot to me on earth.* And now that she is gone, my worldly loss is perfect."

How many a poor fellow would be saved from suicide, from the penitentiary and the gallows every year, had he been blessed with such a wife.

"She made home the pleasantest spot to me on earth." What a grand tribute to that woman's love, and piety, and common sense. Rather different was the testimony of an old man some three years ago, just before he was hung in the Tombs' yard of this city. "I didn't intend to kill my wife, but she was a very aggravating woman." Let each wife inquire, "Which am I?"

